# One or More Species of Pacific Tree Frogs? Insights From Vocal Sexual Signals

**DOI:** 10.1002/ece3.72292

**Published:** 2025-10-08

**Authors:** Alejandro Vélez, Colleen M. Ingram

**Affiliations:** ^1^ Department of Psychology and Neuroscience University of Tennessee Knoxville Tennessee USA; ^2^ Collaborative for Animal Behavior (CoLAB) University of Tennessee Knoxville Tennessee USA; ^3^ Department of Biology San Francisco State University San Francisco California USA; ^4^ Division of Vertebrate Zoology American Museum of Natural History New York New York USA

**Keywords:** acoustic communication, Pacific chorus frogs, reproductive character displacement, sexual selection

## Abstract

Evolutionary divergence in sexual signals may lead to or maintain reproductive isolation between populations. Both selective forces—such as ecological and sexual selection—and random processes like genetic drift may influence the diversification of sexual signals. Understanding the patterns and sources of intraspecific variation in sexual signals among populations can inform the stages of differentiation and speciation. In this study, we investigated patterns of geographic variation in vocal sexual signals and how they relate to genetic and environmental distances among nine populations of Pacific tree frogs. Importantly, the taxonomy of Pacific tree frogs remains unresolved; while some authors recognize only one species ( *Pseudacris regilla* ), other authors propose three distinct species based on mitochondrial DNA lineages ( *P. regilla* , *P. sierra* , and *P. hypochondriaca* ). Our genetic analyses revealed that the nine populations studied span two of the three mitochondrial lineages of Pacific tree frogs. We found that variation in the advertisement calls is better explained by mitochondrial lineage than by geographic or environmental distances between populations. The acoustic properties that have diverged the most between lineages relate to the number of pulses in the call and the rate at which the pulses are delivered. Interestingly, these acoustic properties are important for species recognition in this and other species of frogs. These findings suggest that differences in the vocal sexual signal may lead to premating reproductive isolation between mitochondrial lineages of Pacific tree frogs.

## Graphical abstract

We investigated patterns of geographic variation in mating calls and how they relate to genetic and environmental distances among nine populations of Pacific tree frogs. We show that variation in the mating calls is better explained by mitochondrial lineage than by geographic or environmental distances among populations. Importantly, the acoustic properties that have diverged the most between lineages are important for species recognition in this and other species of frogs, suggesting that differences in the sexual signal may lead to premating reproductive isolation between mitochondrial lineages of Pacific tree frogs.

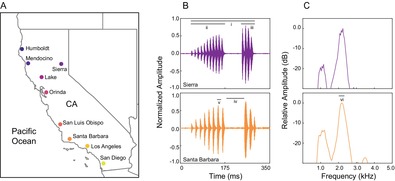

## 1 Introduction

A fundamental goal in evolutionary biology is to understand the factors that generate and maintain biodiversity. Character variation, divergence of characters among populations, and subsequent reproductive isolation are essential stages in the process of speciation (Coyne and Orr 2004). Understanding the interaction and relative contribution of drift, natural selection, and particularly sexual selection in character divergence and speciation has been a topic of great interest in the last couple of decades (Van Doorn et al. 2009; Martin and Mendelson 2012; Wagner et al. 2012; Safran et al. 2013; Servedio and Boughman 2017). Regardless of the extent to which sexual selection pressures drive the process of speciation, sexual isolation has long been considered one of the most important causes of premating reproductive isolation among populations (Mayr 1963; Panhuis et al. 2001; Ritchie 2007; Maan and Seehausen 2011). Thus, differences in sexual displays are widely accepted as crucial in the maintenance of reproductive isolation between species.

Acoustic communication signals mediate mating behaviors across a wide range of taxa, including insects, fish, amphibians, birds, and mammals (Gerhardt and Huber 2002; Marler and Slabbekoorn 2004; Bass and Ladich 2008; Janik 2009). Comparative studies suggest that acoustic signal variation may play an important role in diversification among rapidly speciating lineages, either by promoting reproductive isolation early in speciation or by reinforcing barriers later in the process (Henry 1994; Mendelson and Shaw 2005, 2012; Seddon et al. 2008; Wilkins et al. 2013). Furthermore, studies of within‐species variation reveal that among‐population differences in vocal sexual signals are common and may influence mate recognition (Pröhl et al. 2006, 2007; Podos 2007; Mendelson and Shaw 2012). Thus, studies on among‐population differences in vocal signals and mating behaviors may help to elucidate the stages and drivers of diversification in species in which mating behaviors rely on acoustic communication.

Anuran amphibians are an ideal model for examining acoustic signal divergence and its role in reproductive isolation. In many species of frogs and toads, males produce advertisement calls that function to attract females and repel other rival males. These advertisement calls are often both necessary and sufficient for species recognition and female mate choice (Gerhardt and Huber 2002). Furthermore, the low dispersal ability of many anuran species makes them especially prone to local divergence in sexual traits (Funk et al. 2005; Guarnizo et al. 2009; Lee et al. 2016). Accordingly, several studies have investigated the extent to which differences in vocal sexual signals correlate with genetic, geographic, and environmental distances (Velásquez et al. 2013; Lee et al. 2016; Nali et al. 2023).

In this study, we investigated patterns of geographic variation in the advertisement call of the Pacific tree frog, *Pseudacris regilla* , a species complex with unresolved taxonomy (Nicholson 2025). Pacific tree frogs have a widespread distribution in western North America, ranging from southern Baja California in Mexico to British Columbia in Canada, and inhabit altitudes from sea level to 3300 m above sea level (Snyder and Jameson 1965; Recuero et al. 2006). Importantly, the taxonomy of Pacific tree frogs remains controversial. Previous phylogenetic analyses based on mitochondrial DNA support the existence of three species: *P. hypochondriaca* in Mexico and Southern California, *P. sierra* in central and northern California, and *P. regilla* from Oregon to Canada (Recuero et al. 2006; Jadin et al. 2021). However, this distinction was not supported by analyses based on nuclear DNA (Barrow et al. 2014; Banker et al. 2020). During the breeding season, female Pacific tree frogs choose their mates based on the vocal sexual signals produced by males (Whitney and Krebs 1975; Straughan 1975; Brenowitz and Rose 1999). To the best of our knowledge, there are no reports for Pacific tree frogs on the use of other types of sexual signals (e.g., chemical, visual). Therefore, studies on patterns of geographic variation in vocal sexual displays and mating behaviors in Pacific tree frogs may help to elucidate the taxonomy of this group.

**FIGURE 1:**
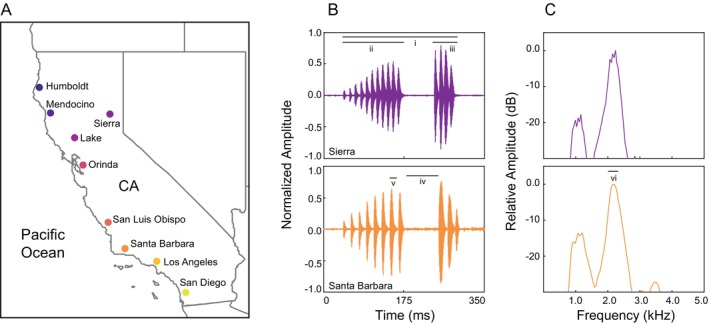
Map of populations sampled labeled by the name of the county where they are located (A). The color gradient from dark blue to yellow reflects the relative location of the populations from north to south. Waveforms (B) and power spectra (C) of representative calls from the Sierra (top) and Santa Barbara (bottom) counties, recorded at water temperatures of 12.0°C and 12.5°C, respectively. Call duration (i) was measured as the time between the onset and the offset of the call, the durations of the first (ii) and second (iii) phase were measured as the time between the onset and the offset of each phase, the number of pulses in each phase were counted, the interphase interval (iv) was measured as the time between the offset of the first phase and the onset of the second phase, pulse rate was calculated as the inverse of the period of the pulse with highest amplitude in phase one (v), call rate was calculated as the inverse of the time between the onset of the call and the onset of the following call (not shown), and the dominant frequency as the peak with highest amplitude in the power spectrum (vi). Power spectra were generated using Hann windows with Fast Fourier Transform (FFT) size of 2048 points and 75% overlap.

Our objectives in this study were to characterize patterns of geographic variation in the advertisement call and to determine the extent to which call properties vary more among than within mitochondrial lineages of Pacific tree frogs. We obtained tissue samples and recorded the calls of male Pacific tree frogs from nine populations in California, USA (Figure 1). If differences in mating calls play a role in premating behavioral isolation between lineages, we predicted larger differences between lineages than within lineages, and stepwise—as opposed to clinal—variation in call properties likely used for species recognition. Studies on Pacific tree frogs and other closely related species suggest that call duration and the rate at which pulses are delivered (i.e., pulse rate) are important traits for discrimination between conspecific and heterospecific males (Straughan 1975; Platz 1989; Lemmon 2009). Furthermore, we have shown that call duration and pulse rate have low within‐ and among‐individual coefficients of variation in Pacific tree frogs (Vélez and Guajardo 2021). In several species of frogs, call properties with low within‐ and among‐individual variation are often under stabilizing selection and important for species recognition, with females showing strong preferences for values close to the population mean (Gerhardt 1991; Gerhardt and Huber 2002). We show here patterns of variation in mating calls that may lead to premating reproductive isolation among mitochondrial lineages of Pacific tree frogs.

## 2 Materials and Methods

### 2.1 Study Subjects and Sites

Our protocols for collecting and handling animals were approved by the Institutional Animal Care and Use Committee at San Francisco State University (Protocol #A17‐07) and conform to guidelines established by the Animal Behavior Society for the use of animals in research. We collected tissue samples and recorded male advertisement calls at nine populations of Pacific tree frogs in California, USA, summarized in Table 1 and Figure 1. Hereafter, we refer to each population by the County (Co) where it is located. Populations were selected to capture ecological and geographical diversity across the species' range, with pairwise distances between sites ranging from ~100 to ~1050 km and the elevation of the sites ranged from close to sea level to almost 2000 m above sea level. Elevational and environmental data for each of our field sites were obtained from the WorldClim database (https://www.worldclim.org/; Hijmans et al. 2005) at a spatial resolution of 2.5′ arcminutes (~5 km^2^), and available in Table A1. Acoustic and genetic data were collected during the breeding season of each population, in times of high chorus activity at night. All the males from which data were collected were found calling in contact with the water.

**TABLE 1:** Geographic coordinates, elevation, and sample sizes of the nine localities.

County	Latitude	Longitude	Elevation (masl)	*N* tissue	*N* recordings
Humboldt	40.687	−124.214	35	5	16
Mendocino	39.718	−123.653	572	5	12
Sierra	39.674	−120.631	1999	4	23
Lake	38.871	−122.419	591	4	8
Orinda	37.930	−122.173	225	2	24
San Luis Obispo	35.530	−121.078	102	4	15
Santa Barbara	34.695	−120.042	373	4	23
Los Angeles	34.098	−118.657	462	4	23
San Diego	33.148	−117.252	94	4	12

### 2.2 Collection and Analyses of Genetic Data

#### 2.2.1 Sample Collection and DNA Extraction

We collected toe clips from the hind legs of adult males. Tissue samples were preserved in 95% ethanol and stored at −20°C until extraction. Genomic DNA was extracted using the QIAamp Fast DNA Tissue Kit (QIAGEN, Valencia, CA, USA) following the manufacturer's protocol. Given the small size of toe clips (< 0.05 g), the entire sample was used for each extraction to maximize yield. To improve total DNA recovery, the elution step was modified to include three sequential elutions of 50 μL each (total 150 μL), using AE buffer. Only the first eluate was used for downstream applications, while the second and third were stored at −20°C for future research.

#### 2.2.2 Marker Selection and PCR Amplification

We amplified a 609‐bp fragment of the mitochondrial cytochrome *b* (*cytb*) gene using primers MVZ15 and MVZ18 (Moritz et al. 1992): MVZ15 (5′‐GAACTAATGGCCCACACWWTACGNAA‐3′) and MVZ18 (5′‐GTCTTTGTATGAGAAGTATG‐3′), targeting a region known for strong phylogenetic signal in *Pseudacris* (Recuero et al. 2006; Lemmon et al. 2007). PCR was conducted in 25 μL reactions using KAPA HiFi HotStart ReadyMix (Roche), with thermocycling conditions: 94°C for 5 min; 35 cycles of 94°C for 30 s, 52°C for 30 s, and 72°C for 1 min; final extension at 72°C for 10 min. Amplification success was verified via agarose gel electrophoresis, and successful products were purified using ExoSAP‐IT.

#### 2.2.3 DNA Sequencing, Alignment, and Phylogenetic Inference

Sanger sequencing was performed bidirectionally on an Applied Biosystems 3730xl DNA Analyzer (96‐capillary platform; Thermo Fisher Scientific) at ELIM Biopharmaceuticals (Hayward, CA, USA). The resulting chromatograms were quality‐checked, and base calls were manually verified. Sequences were queried against the GenBank nucleotide database using BLASTn (Altschul et al. 1990) via the NCBI web interface (https://blast.ncbi.nlm.nih.gov/) to confirm sequence identity. Reference sequences were downloaded from GenBank (Benson et al. 2018). Multiple sequence alignments were performed using MUSCLE v5.1 (Edgar 2004). All newly obtained sequences were deposited in GenBank under accession numbers PV685967–PV685997.

### 2.3 Acoustic Recordings and Analyses

Digital recordings (44.1 kHz, 16‐bit resolution) of male advertisement calls were obtained using a TASCAM D‐40 hand‐held recorder coupled with a Sennheiser ME‐66 microphone (Table 1). We placed the tip of the microphone in front of the focal male, at approximately 50–75 cm, and we recorded vocal activity for about 2 min. After completing the recording, we captured the male by hand and measured its snout‐to‐vent length (SVL) to the nearest 0.1 mm with dial calipers and its mass to the nearest 0.1 g with a Pesola spring scale. We used high‐precision thermometers (H‐B Instrument Enviro‐Safe or Fisher Brand Traceable thermometers) to measure the temperature of the water to the nearest 0.1°C.

From each recording, we selected a set of four consecutive diphasic calls with no overlapping calls from other neighboring males. We used Raven Pro v1.5 to measure eight temporal properties and one spectral property from the first three calls in the set (Figure 1). All temporal properties were measured in the waveform display, and the spectral property was measured in the selection spectrum display. Call duration was measured as the time between the onset and the offset of the call. The instantaneous call rate was calculated as the inverse of the call period, which was measured from the onset of the call to the onset of the subsequent call. For each of the two phases of the call separately, we counted the number of pulses in the phase, and we determined phase duration by measuring the time between the onset of the first pulse and the offset of the last pulse in the phase. The interphase interval was measured as the time between the offset of the last pulse of the first phase and the onset of the first pulse of the second phase. We calculated instantaneous pulse rate as the inverse of the period of the pulse with the highest amplitude in phase 1 (Vélez and Guajardo 2021). Finally, we generated an average power spectrum of the entire call using Hann windows with Fast Fourier Transform (FFT) size of 2048 points and 75% overlap and measured the dominant frequency of the call as the spectral peak with the highest relative amplitude. For each call property, we used the median of the three calls analyzed as the data contributed by each male on all subsequent analyses.

**FIGURE A1:**
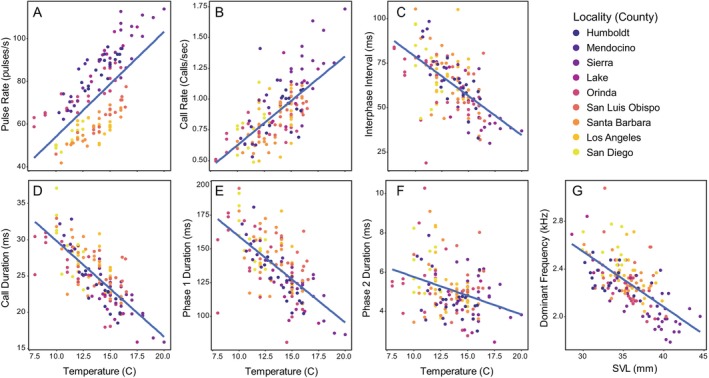
Correlations between temperature and pulse rate (A), call rate (B), interphase interval (C), call duration (D), duration of phase 1 (E), and duration of phase 2 (F), and between SVL and dominant frequency (G). Lines in all panels depict significant correlations between the variables.

In Pacific tree frogs, temperature influences the rate at which pulses and calls are produced, as well as the duration of each phase, of the interphase interval, and of the entire call (Vélez and Guajardo 2021; Figure A1). Similarly, call dominant frequency is negatively correlated with the subject's SVL (Vélez and Guajardo 2021; Figure A1). Accordingly, we standardized temporal call properties to a common temperature of 14°C and call dominant frequency to a common SVL of 35 mm following Platz and Forester (1988). We chose these values because they are close to the mean temperature at which we recorded calls and the mean SVL measured from recorded individuals, and because they have been used in several studies of frogs from the genus *Pseudacris* (Platz and Forester 1988; Platz 1989; Lemmon 2009; Bee et al. 2010; Vélez and Guajardo 2021; Messersmith et al. 2024). We performed all subsequent analyses using temperature‐ and SVL‐corrected values.

### 2.4 Statistical Analyses

#### 2.4.1 Phylogenetic Reconstruction and Genetic Distances

Prior to phylogenetic analysis, the nucleotide substitution model was determined using jModelTest 2.1.10 (Darriba et al. 2012) via the CIPRES Science Gateway (Miller et al. 2010). A total of 88 candidate models were evaluated under both the Akaike Information Criterion (AIC) and the Bayesian Information Criterion (BIC). Both criteria selected the TrN + I + G model (Tamura–Nei with a proportion of invariant sites and gamma‐distributed rate heterogeneity) as the best fit for the dataset.

As the TrN model is not implemented in many phylogenetic analysis programs, the GTR + I + Γ model (General Time Reversible with invariant sites and gamma distribution) was used for all downstream analyses. This substitution is common in phylogenetic workflows and provides a more general, flexible model framework, albeit with the potential for slight overparameterization.

Maximum likelihood (ML) analysis was conducted in RAxML v8.2.12 (Stamatakis 2014) via the Geneious plugin, using the GTR + I + Γ model. Five independent ML searches were run with unique random seeds to enhance tree space exploration. The best‐scoring tree (highest likelihood) was retained, and 1000 rapid bootstrap replicates were performed to evaluate node support.

Bayesian inference was performed using MrBayes v3.2 (Ronquist et al. 2012), also applying the GTR + I + Γ model. Two independent MCMC runs were conducted, each with four chains for 1 million generations, sampling every 1000 generations. Convergence was assessed by ensuring the average standard deviation of split frequencies (ASDSF) was < 0.01 and effective sample sizes (ESS) > 200 for all parameters, as verified in Tracer v1.7 (Rambaut et al. 2018). The first 25% of sampled trees were discarded as burn‐in, and the remaining trees were used to generate a majority‐rule consensus tree with posterior probabilities representing clade support.

To assess evolutionary divergence among populations, patristic distances were calculated from the ML tree. These distances represent the sum of branch lengths separating two taxa, providing a tree‐informed measure of genetic divergence (Felsenstein 2004). Mean patristic distances between defined population clades were extracted using Geneious Prime, enabling comparisons of genetic structure across populations.

#### 2.4.2 Call Variation Between and Within Mitochondrial Lineages

All statistical analyses were conducted in R v4.4.1 (R Core Team 2024). To assess whether variation in acoustic traits reflects underlying phylogenetic relationships, we tested for phylogenetic signal using Pagel's *λ* using the package phytools (Revell 2024). Because Pagel's *λ* requires fully resolved trees with monophyletic tips, we pruned our ML phylogeny to include a single representative per population, collapsing branches where tips did not form monophyletic groups (available as Supporting Information). Phylogenetic signal for each acoustic trait was tested using the mean value at each population.

To determine the extent to which call traits vary between mitochondrial lineages and among populations within lineages (see Section 3), we performed ANOVAs with population nested within lineage. To account for multiple comparisons, we used an alpha level of 0.0056 based on a Bonferroni correction for nine call traits. As a measure of effect size, we report partial *η* ^2^ values calculated using the package effectsize v0.8.8 (Ben‐Shachar et al. 2020).

#### 2.4.3 Geographic, Genetic, and Environmental Correlates of Call Divergence

We used the geosphere package v1.5‐20 (Hijmans 2024) in R to calculate the pairwise Haversine distances between populations. We first explored isolation by distance (IBD) by testing the correlation between geographic and genetic distances using a Mantel test with 9999 permutations using the vegan package v2.6‐8 (Oksanen et al. 2024) in R. It is important to note that our dataset includes samples from two mitochondrial lineages of Pacific tree frogs (see Section 3), and that the existence of genetic clusters or a hierarchical structure among populations may bias results from simple Mantel tests (Meirmans et al. 2011; Meirmans 2012). To account for the hierarchical structure of our dataset, we used a partial Mantel test including a binary model matrix coding for population comparisons within (0) and between (1) mitochondrial lineages (Rodrigues et al. 2002; Meirmans et al. 2011; Picq et al. 2020).

To calculate the acoustic distances between populations, we first ran a principal components analysis (PCA) using the nine variables measured from the advertisement calls. We then used the first three principal components (PCs; see Section 3) and calculated pairwise Euclidean distances between centroids in PC space of each population using the vegan package v2.6‐8 (Oksanen et al. 2024) in R. We used simple Mantel tests and partial Mantel tests with our binary model matrix as a covariate to investigate the relationship between signal divergence and genetic and geographic distances.

Similarly, to calculate environmental distances between populations, we ran a PCA including elevation and the 19 bioclimatic variables of each population obtained from the WorldClim database (Table A1). All environmental variables were log‐transformed; prior to transformation, all temperature variables were converted from Celsius to Kelvin (to avoid negative values), and precipitation variables that included zero values were adjusted by adding one. We then used the first three PCs to calculate pairwise Euclidean distances between populations. To explore isolation by environment (IBE), we tested the correlation between environmental and genetic distances using a simple and a partial mantel test that included the binary model matrix as a covariate. We then investigated the relationship between environmental distance and signal divergence with a simple and a partial Mantel test including the binary model matrix.

## 3 Results

### 3.1 Patterns of Genetic Variation

**FIGURE A2:**
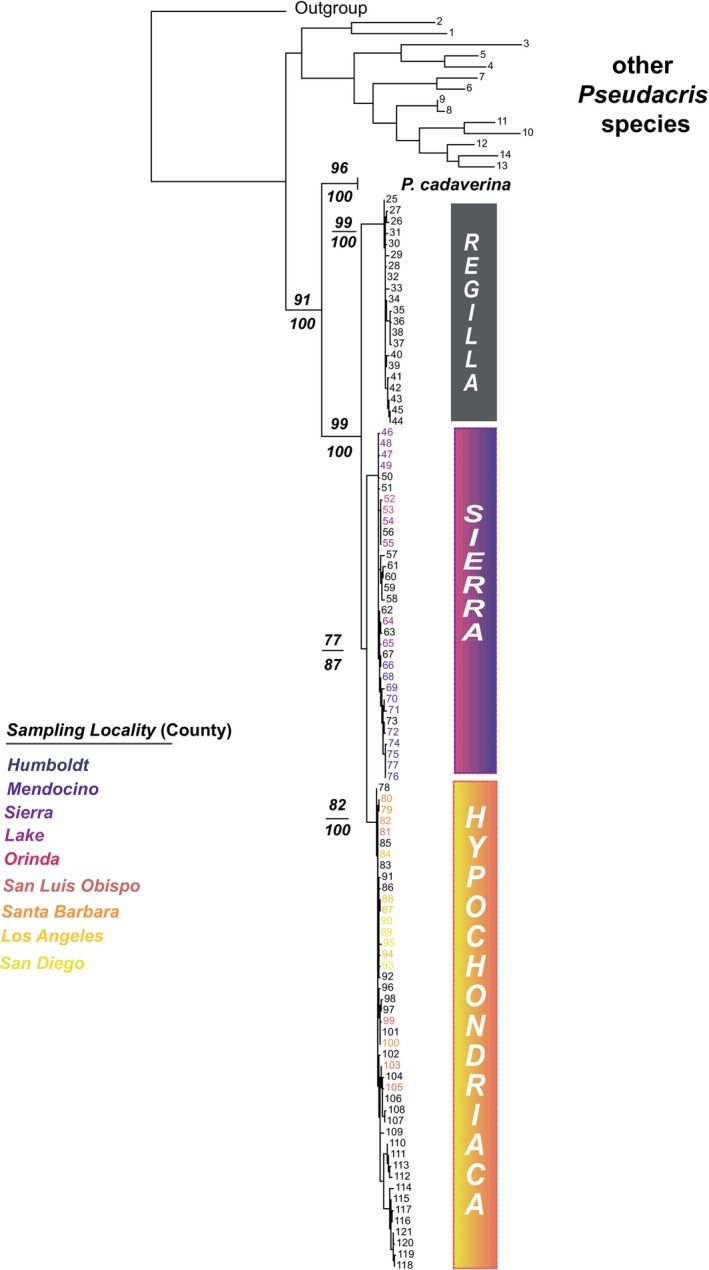
Maximum likelihood phylogeny of cytochrome *b* sequences from 122 taxa, including representatives of all recognized *Pseudacris* species and outgroup taxon *Acris gryllus* , inferred using RAxML under the GTR + I + Γ model. Sequences include both newly generated data (36 samples from 9 populations) and curated sequences from previous studies, trimmed to overlapping regions. Bootstrap support values (RAxML) and posterior probabilities (MrBayes) are shown for major nodes. This comprehensive tree provides the broader phylogenetic context for Figure 2, which presents a pruned subset focused on the *Pseudacris regilla* species group. Tips are labeled with sample numbers corresponding to Table A2, newly generated sequences are color‐coded by population to match the legend, and black labels depict previously published sequences from different localities.

A total of 36 tree frog samples were successfully extracted, sequenced, and included in phylogenetic analyses, representing nine geographically and ecologically diverse localities across California (Figure 1; Table 1). Sampling spanned a broad latitudinal and elevational gradient, from 94 m at San Diego Co to 1999 m at Sierra Co. Sample sizes per site ranged from 2 to 5 individuals, with Humboldt Co and Mendocino Co contributing the largest samples (*n* = 5 each). Sequences were aligned with a curated dataset of 86 previously published *cytb* sequences, yielding a final dataset of 121 *Pseudacris* and one outgroup taxon ( *Acris gryllus* ; Figure A2; Table A2).

**FIGURE 2:**
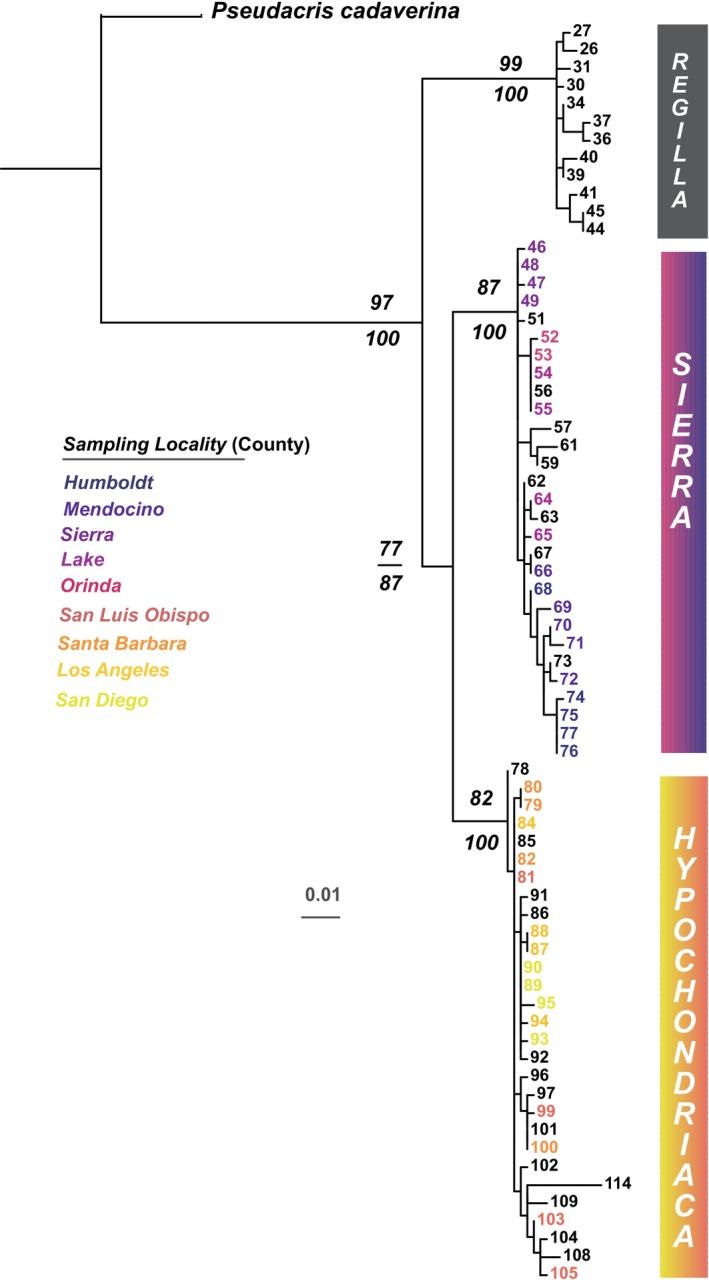
Maximum likelihood phylogeny of *Pseudacris regilla* species group based on cytochrome *b* sequences, inferred using RAxML and PhyML under the GTR + I + Γ substitution model. The tree is a pruned subset of a larger phylogeny (122 taxa; see Figure A2) that included all recognized *Pseudacris* species and used *Acris* as an outgroup. This figure focuses on 36 newly sequenced individuals from nine localities, alongside previously published sequences curated for overlapping regions. *P. cadaverina* was used to root this tree. Support values on major nodes represent bootstrap proportions (RAxML) and posterior probabilities (MrBayes). Tips are labeled with sample numbers corresponding to Table A2, newly generated sequences are color‐coded by population to match the legend, and black labels depict previously published sequences from different localities. All methods recovered consistent relationships among major clades, though genetic structuring within clades was limited. Names in color bars represent the three mitochondrial lineages described for *P. regilla* .

Maximum likelihood (ML) and Bayesian inference (BI) analyses yielded largely congruent topologies, supporting the three major clades within the *P. regilla* complex. The ML tree (inferred in RAxML under the GTR + I + Γ model) recovered a monophyletic “regilla” group with strong support for three major lineages corresponding to previously described clades. The nine focal populations were divided between a northern *P. sierra* clade (Humboldt Co, Mendocino Co, Sierra Co, Lake Co, Orinda Co) and a southern *P. hypochondriaca* clade (San Luis Obispo Co, Santa Barbara Co, Los Angeles Co, San Diego Co). Clade‐level support was strong, with ML bootstrap values ≥ 85% and posterior probabilities ≥ 0.95 at key nodes (Figure 2).

The geographic boundary between these clades lies south of the Orinda Co site, the southernmost population in the *P. sierra* clade sampled. Despite the wide geographic distribution within the *P. sierra* group, intra‐clade patristic distances were relatively low (mean = 0.0085). For example, pairwise distances between Humboldt Co and the other northern sites ranged from 0.0096 (Mendocino Co) to 0.0150 (Orinda Co). Notably, the genetic distance between Sierra Co (a high‐elevation site) and Orinda Co was just 0.00875, and between Sierra Co and Lake Co was 0.00750, indicating modest divergence despite substantial topographic separation, suggesting recent divergence or ongoing gene flow. In contrast, pairwise distances between northern and southern populations (between clades) were considerably higher, ranging from 0.03688 to 0.04600. For example, genetic divergence between Orinda Co and San Luis Obispo Co was 0.04125, while Humboldt Co and San Luis Obispo Co differed by 0.04600. These values support the presence of a phylogeographic break and long‐term evolutionary separation between the *P. sierra* and *P. hypochondriaca* clades.

Patristic distance analyses further revealed clear genetic structuring across populations. Mean pairwise distances from the ML tree indicated moderate to high divergence between localities, with the highest values observed between northern coastal populations (e.g., Humboldt Co) and southern interior sites (e.g., Los Angeles Co, San Diego Co). Conversely, within‐locality divergence was minimal (typically < 0.01), reflecting high population‐level genetic cohesion.

Geographic clustering was especially pronounced among northern and high‐elevation populations, consistent with long‐term isolation or limited gene flow. The distinctiveness of high‐elevation populations such as Sierra Co may reflect historical isolation and potential adaptation to montane environments (Morrison and Hero 2003).

Among southern *P. hypochondriaca* populations, genetic distances were low, suggesting recent common ancestry or continued connectivity. For example, San Diego Co and Los Angeles Co were separated by a patristic distance of only 0.0025, while San Luis Obispo Co and Santa Barbara Co differed by 0.0069 (Table 2). These findings reinforce prior evidence for significant genetic structuring within the *P. regilla* complex across California.

**TABLE 2:** Pairwise patristic genetic distances among Pacific tree frog localities. Patristic distances were calculated as the sum of branch lengths separating each pair of localities on a maximum likelihood tree generated in RAxML using the GTR + I + Γ substitution model, based on a partial sequence of the mitochondrial gene cytochrome *b*. Values represent substitutions per site. Boldface values highlight distances between localities assigned to different mitochondrial clades (sierra vs. hypochondriaca), indicating deeper genetic divergence.

	Humboldt	Mendocino	Sierra	Lake	Orinda	San Luis Obispo	Santa Barbara	Los Angeles	San Diego
Humboldt	0.0040								
Mendocino	0.0096	0.0090							
Sierra	0.0100	0.0100	0.0000						
Lake	0.0100	0.0100	0.0075	0.0067					
Orinda	0.0150	0.0130	0.0088	0.0050	0.0000				
San Luis Obispo	**0.0460**	**0.0460**	**0.0381**	**0.0400**	**0.0413**	0.0083			
Santa Barbara	**0.0430**	**0.0420**	**0.0369**	**0.0400**	**0.0400**	0.0069	0.0033		
Los Angeles	**0.0460**	**0.0430**	**0.0381**	**0.0400**	**0.0400**	0.0075	0.0056	0.0000	
San Diego	**0.0450**	**0.0440**	**0.0388**	**0.0400**	**0.0400**	0.0081	0.0056	0.0025	0.0017

### 3.2 Patterns of Call Variation Between and Within Mitochondrial Lineages

Mean call properties for each mitochondrial lineage and population are summarized in Table 3. We found significant phylogenetic signal in pulse rate (*λ* = 0.992, *p* < 0.0001) and the number of pulses in phase 1 (*λ* = 0.684, *p* = 0.016). No significant phylogenetic signal was detected in the remaining acoustic traits (all *λ* < 0.5; all *p* > 0.13).

**TABLE 3:** Mean ± SD call properties for each mitochondrial lineage (lin.) and locality (listed by county name), standardized to a common temperature of 14°C and a common SVL of 35 mm.

	*N* pulses Phase 1	*N* pulses Phase 2	Pulse rate (pulses/s)	Call Dur (ms)	Phase 1 Dur (ms)	Phase 2 Dur (ms)	IPI Dur (ms)	Call rate (calls/s)	Dominant frequency (Hz)
Sierra lin.	11.3 ± 1.2	4.1 ± 1.1	84.3 ± 6.5	236.9 ± 20.3	128.6 ± 15.0	48.4 ± 11.8	59.1 ± 11.6	0.95 ± 0.17	2250.3 ± 125.6
Hypochondriaca lin.	9.1 ± 0.9	3.5 ± 0.8	61.5 ± 5.3	255.0 ± 24.5	139.7 ± 15.9	51.6 ± 12.3	63.1 ± 13.3	0.86 ± 0.13	2385.9 ± 168.0
Humboldt	11.1 ± 0.6	3.8 ± 0.7	84.2 ± 3.8	229.8 ± 13.6	124.9 ± 7.5	45.3 ± 7.0	60.4 ± 8.7	0.88 ± 0.14	2236.9 ± 111.4
Mendocino	11.5 ± 0.7	3.4 ± 1.1	86.3 ± 6.5	252.1 ± 19.8	135.8 ± 11.9	46.9 ± 13.9	69.6 ± 8.9	0.97 ± 0.21	2328.8 ± 144.2
Sierra	12.2 ± 1.0	4.5 ± 0.9	85.3 ± 7.3	247.4 ± 14.4	135.7 ± 9.7	52.3 ± 8.6	57.9 ± 9.2	1.07 ± 0.16	2183.3 ± 110.6
Lake	11.0 ± 0.5	3.7 ± 1.1	89.0 ± 4.0	220.8 ± 12.4	118.2 ± 8.6	42.3 ± 10.1	59.0 ± 10.6	0.94 ± 0.18	2331.4 ± 138.6
Orinda	10.5 ± 1.5	4.3 ± 1.3	81.0 ± 6.5	229.3 ± 22.5	124.1 ± 21.0	49.5 ± 15.3	54.1 ± 13.7	0.88 ± 0.13	2257.0 ± 102.4
San Luis Obispo	9.9 ± 0.5	3.8 ± 0.9	60.7 ± 3.1	265.9 ± 21.9	150.4 ± 13.1	55.1 ± 13.7	62.0 ± 12.6	0.81 ± 0.06	2436.0 ± 243.3
Santa Barbara	8.9 ± 0.9	3.3 ± 0.7	61.2 ± 3.6	249.2 ± 24.6	138.1 ± 17.0	48.2 ± 12.7	62.6 ± 13.0	0.85 ± 0.10	2317.4 ± 99.8
Los Angeles	8.7 ± 0.8	3.4 ± 0.7	59.5 ± 7.0	254.8 ± 21.4	135.1 ± 15.1	51.8 ± 10.8	65.8 ± 13.3	0.87 ± 0.17	2382.8 ± 149.7
San Diego	9 ± 0.6	3.5 ± 0.6	67 ± 2.4	253.1 ± 31.2	138.1 ± 13.8	53.1 ± 12.1	60.4 ± 15.4	0.94 ± 0.15	2460.5 ± 158.8

Abbreviations: Dur, duration; IPI, interphase interval.

**FIGURE 3:**
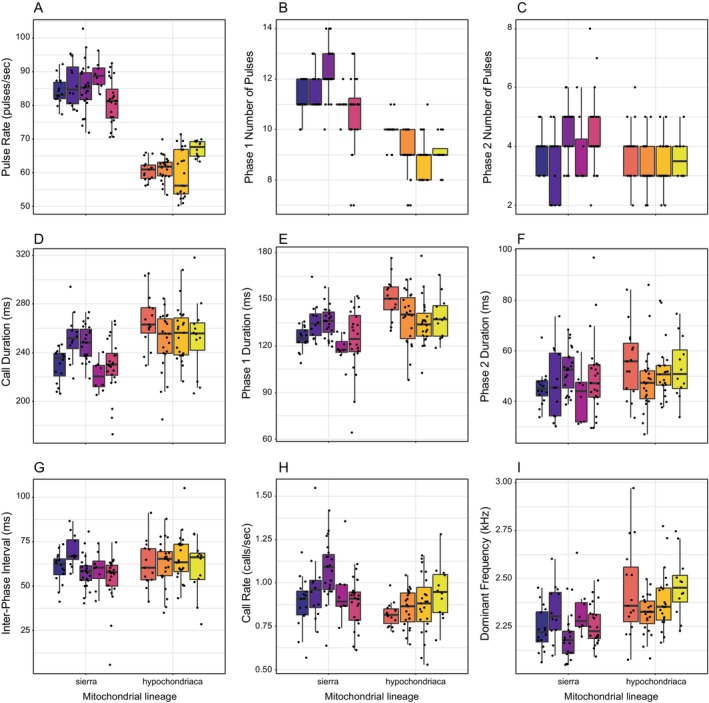
Differences among populations nested within mitochondrial lineages in pulse rate (A), number of pulses in phase one (B), number of pulses in phase two (C), call duration (D), duration of phase one (E), duration of phase two (F), interphase interval (G), call rate (H), and dominant frequency (I). Raw data (dots), median (horizontal line), interquartile range (box), and 1.5 times interquartile range (whiskers) values of each call property for males from each population, ordered horizontally from north to south and grouped by mitochondrial lineage.

Results from our statistical analyses comparing the nine call properties among mitochondrial lineages and among sites within lineage are summarized in Table 4 and Figure 3. As evidenced by large effect sizes (ES ≥ 0.26; Cohen 1992), the most different call properties between mitochondrial lineages were pulse rate (*F*_1,147_ = 647.51, *p* < 0.0001, partial *η* ^2^ = 0.81), and the number of pulses in phase 1 (*F*_1,147_ = 198.77, *p* < 0.0001, partial *η* ^2^ = 0.57). We also found significant differences between mitochondrial lineages in call dominant frequency and call duration with medium effect sizes (0.13 ≤ ES < 0.26; Cohen 1992) and, with small effect sizes (ES < 0.13; Cohen 1992), in the duration of phase 1, the number of pulses in phase 2, and call rate. Within mitochondrial lineages, we found significant differences among populations in the same call properties, except for the number of pulses in phase 2. All acoustic properties that varied significantly among populations had a medium effect size.

**TABLE 4:** Results from nested ANOVAs on call properties ordered by descending effect size between lineages. Values in boldface highlight significant effects (significance criterion: *α* = 0.0056).

	Lineage			Population within lineage		
	*F*_1,147_	Pr(> *F*)	Partial eta^2^	*F*_7,147_	Pr(> *F*)	Partial eta^2^
Pulse rate	**647.51**	**< 0.0001**	**0.81**	**4.45**	**0.0002**	**0.17**
Phase 1 number of pulses	**198.77**	**< 0.0001**	**0.57**	**7.14**	**< 0.0001**	**0.25**
Dominant frequency	**36.46**	**< 0.0001**	**0.20**	**3.26**	**0.0031**	**0.13**
Call duration	**28.66**	**< 0.0001**	**0.16**	**3.91**	**0.0006**	**0.16**
Phase 1 duration	**22.49**	**< 0.0001**	**0.13**	**3.80**	**0.0008**	**0.15**
Phase 2 number of pulses	**15.73**	**0.0001**	**0.10**	2.51	0.0182	0.11
Call rate	**13.58**	**0.0003**	**0.08**	**4.19**	**0.0003**	**0.17**
Interphase interval duration	4.35	0.0387	0.03	2.18	0.0389	0.09
Phase 2 duration	2.75	0.0995	0.02	1.34	0.2352	0.06

### 3.3 Relationships Between Genetic, Geographic, Environmental, and Acoustic Distances

A PCA on the nine call properties revealed three PCs with eigenvalues > 1.0, which together explain 75% of the total variance (Table 5). The first PC (PC1) explained 30% of the variance and loaded heavily (≥ 0.4) and negatively on pulse rate. Explaining 26% of the variance, PC2 loaded heavily and negatively on the duration of the call and of each phase. PC3 explained an additional 19% of the variance and loaded heavily and negatively on the number of pulses in phase 1 and the interphase interval. These three PCs were used to calculate Euclidian distances in acoustic PC space between pairs of populations. Similarly, environmental variables, including the elevation and the 19 bioclimatic variables, can be summarized by three PCs with eigenvalues > 1.0 that describe 94% of the variation (Table 6). Using these three PCs, environmental distance between pairs of populations was calculated as the Euclidean distance between the centroids in environmental space.

**TABLE 5:** Loadings of call properties on the three PCs with eigenvalues larger than 1.0 and proportion of variance explained by each PC. Values in boldface highlight loadings with absolute values > 0.4.

	PC1	PC2	PC3
Pulse rate	**−0.52**	0.10	−0.23
Phase 2 duration	−0.08	**−0.53**	0.36
Phase 1 duration	0.22	**−0.47**	−0.35
Call duration	0.29	**−0.51**	−0.27
Phase 1 N pulses	−0.39	−0.23	**−0.44**
IPI duration	0.36	0.15	**−0.43**
Dominant frequency	0.32	−0.09	0.21
Call rate	−0.31	−0.06	−0.29
Phase 2 N pulses	−0.34	−0.38	0.33
Eigenvalue	2.66	2.37	1.68
Proportion of variance	0.30	0.26	0.19
Cumulative proportion	0.30	0.56	0.75

**TABLE 6:** Loadings of climatic variables on the three PCs with eigenvalues larger than 1.0 and proportion of variance explained by each PC.

	PC1	PC2	PC3
BIO‐01: Annual mean temperature	0.28	−0.01	0.15
BIO‐02: Mean diurnal range	0.00	−0.38	0.15
BIO‐03: Isothermality	0.18	0.24	0.22
BIO‐04: Temperature seasonality	−0.12	−0.38	−0.02
BIO‐05: Max temperature of warmest month	0.10	−0.38	0.16
BIO‐06: Min temperature of coldest month	0.25	0.17	0.19
BIO‐07: Temperature annual range	−0.08	−0.41	0.00
BIO‐08: Mean temperature of wettest quarter	0.27	0.12	0.15
BIO‐09: Mean temperature of driest quarter	0.21	−0.25	0.14
BIO‐10: Mean temperature of warmest quarter	0.21	−0.25	0.11
BIO‐11: Mean temperature of coldest quarter	0.27	0.12	0.14
BIO‐12: Annual precipitation	−0.26	0.06	0.35
BIO‐13: Precipitation of wettest month	−0.25	0.04	0.42
BIO‐14: Precipitation of driest month	−0.27	0.05	−0.14
BIO‐15: Precipitation seasonality	0.24	−0.09	0.34
BIO‐16: Precipitation of wettest quarter	−0.25	0.06	0.41
BIO‐17: Precipitation of driest quarter	−0.28	0.08	−0.03
BIO‐18: Precipitation of warmest quarter	−0.28	0.09	0.06
BIO‐19: Precipitation of coldest quarter	−0.25	0.04	0.41
Elevation	−0.13	−0.36	−0.03
Eigenvalue	11.66	5.54	1.56
Proportion of variance	0.58	0.28	0.08
Cumulative proportion	0.58	0.86	0.94

**FIGURE 4:**
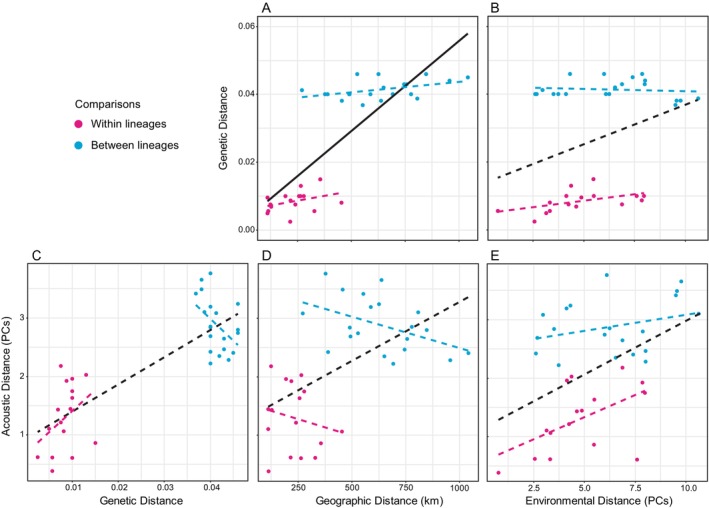
Population pairwise comparisons between genetic and geographic distances (A), genetic and environmental distances (B), and acoustic distance and genetic (C), geographic (D), and environmental (E) distances. The black solid line in (A) represents significant simple and partial Mantel tests, while the dotted black lines in (B) through (E) represent significant simple Mantel tests, but not partial Mantel tests. The dotted magenta and cyan lines are used for visual inspection of the relationship between variables when comparing populations within and between mitochondrial lineages, respectively.

We found a significant correlation between genetic and geographic distance with both our simple Mantel test and our partial Mantel test controlling for comparisons within and between mitochondrial lineages (Table 7; Figure 4A). Genetic and environmental distances were significantly correlated using a simple Mantel test but not using a partial Mantel test controlling for comparisons between and within populations (Table 7; Figure 4B). Visual inspection reveals little relationship between genetic and environmental distance when comparing populations between mitochondrial lineages.

**TABLE 7:** Results from simple Mantel tests and partial Mantel tests including a binary model matrix coding for population comparisons within (0) and between (1) mitochondrial lineages. Values in boldface highlight significant effects.

Correlation	Mantel		Partial Mantel	
	*r*	*p*	*r*	*p*
Genetic—Geographic	**0.820**	**< 0.0001**	**0.369**	**0.0281**
Call—Genetic	**0.698**	**0.0091**	−0.183	0.8840
Call—Geographic	**0.618**	**0.0096**	−0.334	0.9836
Genetic—Environmental	**0.316**	**0.0366**	0.143	0.2583
Call—Environmental	**0.397**	**0.0132**	0.311	0.1049

Call distance was significantly correlated with genetic distance using a simple Mantel test, but not when controlling for comparisons between and within lineages (Table 7; Figure 4C). Visual inspection of the relationship between call and genetic distance suggests that call distance increases with genetic distance within lineages, but not when comparing populations between lineages (Figure 4C). Similarly, call distance was significantly correlated with geographic distance, but not when controlling for comparisons between and within lineages (Table 7; Figure 4D). Visual inspection reveals a weak negative relationship between call and geographic distance both within and between lineages (Figure 4D). Call distances were also correlated with environmental distance with the simple Mantel test, but not when controlling for between‐ and within‐lineage comparisons, and visual inspection reveals a weak relationship between call and environmental distances between populations of different mitochondrial lineages (Table 7; Figure 4E).

## 4 Discussion

The objectives of this study were to characterize patterns of geographic variation in the vocal sexual signal and to determine the extent to which vocal sexual signals vary among mitochondrial lineages of Pacific tree frogs. Results from our genetic analysis confirm the genetic structure previously reported for mitochondrial genes in this group and reveal that our dataset spanned two of the three mitochondrial lineages of Pacific tree frogs. This pattern mirrors findings from other California taxa, including *Ensatina* salamanders (patristic divergence 0.02–0.05; Wake 2006), Pacific newts (*Taricha*, 0.03–0.04; Kuchta and Tan 2005), and the California red‐legged frog ( *Rana draytonii* , 0.03–0.05; Shaffer et al. 2004), all of which show deep genetic breaks across central California attributed to isolation in coastal and interior refugia. These examples suggest a common biogeographic history across diverse taxa, with glacially mediated barriers fragmenting populations and promoting allopatric divergence (Hewitt 2000; Zamudio and Wieczorek 2007; Rissler and Apodaca 2007). Importantly, our acoustic analyses revealed strong differences between mitochondrial lineages in the advertisement call, particularly in the rate at which pulses are delivered and in the number of pulses. Furthermore, our results suggest that differences in the advertisement calls are better explained by mitochondrial lineage than by geographic or environmental distances between populations. Indeed, the acoustic properties that have diverged most between lineages—the number of pulses in the call and the rate at which pulses are delivered—also exhibit significant phylogenetic signal, suggesting these traits evolved in concert with the historical lineage split.

Together, these results support a scenario where long‐term isolation in Pleistocene refugia not only drove genetic divergence but may have also promoted reproductive isolation through signal divergence—a pattern observed in other taxa including *Rana* frogs (Fouquet et al. 2007), *Hyla* tree frogs (Lemmon 2009), and Neotropical species (Guerra and Ron 2008; Velásquez et al. 2013), where historical isolation correlates with divergence in advertisement calls.

A first step towards understanding the factors that shape the evolution of sexual signals is to test the null hypothesis that divergence in such traits is correlated with genetic divergence and geographic distance between populations (Panhuis et al. 2001). Support for this null hypothesis has been reported for some species of frogs (Funk et al. 2005; Pröhl et al. 2007; Amézquita et al. 2009; Velásquez et al. 2013; Lee et al. 2016). Our results show evidence for genetic drift, as we found positive relationships between genetic and geographic distances in our entire dataset and when comparing populations between and within mitochondrial lineages. Importantly, we found a correlation between genetic and mating‐call distances when comparing pairs of populations within mitochondrial lineages. However, comparisons of populations from different mitochondrial lineages did not show a positive relationship between genetic and call distances. Additionally, geographic distance was not positively correlated with mating‐call distance when comparing populations within or between mitochondrial lineages. Together, these results suggest that the evolution of vocal sexual signals in Pacific tree frogs cannot be fully explained by random factors, like drift.

Variation in sexual signals not explained by drift is often hypothesized to be due to ecological or sexual selection. Indeed, several studies have investigated the extent to which ecological processes and environmental differences influence the evolution of sexual signals in frogs and other taxa (Guerra and Ron 2008; Martin and Mendelson 2012; Lee et al. 2016). Our results do not provide support for isolation by environment in Pacific tree frogs, as we did not find a correlation between genetic and environmental distance when controlling for comparisons within and between mitochondrial lineages. In addition, divergence in mating calls was also not correlated with environmental distance when controlling for between‐ and within‐lineage comparisons. These results suggest that the environmental factors that we included in our analyses have little effect on genetic divergence and on the evolution of vocal sexual signals in Pacific tree frogs.

Our results show strong differences in the vocal sexual signal of Pacific tree frogs between the “*hypochondriaca*” mitochondrial lineage in Southern California and the “*sierra*” lineage in Central and Northern California. Snyder and Jameson (1965) studied variation in Pacific tree frog calls along a broader geographic range, from Canada to Mexico. Interestingly, their results suggest that calls from populations in Central California and northward differ strongly from those in Southern California and Mexico. In their study, call dominant frequency was the property that varied the most among populations, and they suggested that differences in spectral call properties may lead to premating behavioral isolation between populations (Snyder and Jameson 1965). In our study, dominant frequency had the third largest effect size when comparing between mitochondrial lineages (Table 4; Figure 3). Snyder and Jameson (1965) did not include the two properties that varied the most between lineages in our study: pulse rate and the number of pulses in the first phase of the call (Table 4; Figure 3). The faster pulse rates we measured in populations of the northern lineage in California (Table 3) are similar to those reported by Rose and Brenowitz (2002) from a Pacific tree frog population located in Washington, USA, north of the northernmost population in our study. Early descriptions of the mating call of Pacific tree frogs from Southern California report a similar number of pulses and pulse rates as the ones we report here for populations from the southern mitochondrial lineage (Littlejohn 1971; Straughan 1975; Figure 3; Table 3). Interestingly, pulse rate and the number of pulses have been considered important for species recognition in *Pseudacris* (Platz 1989; Lemmon 2009), and in other species of tree frogs (Loftus‐Hills and Littlejohn 1971; Bush et al. 2002), suggesting that call differences between the mitochondrial lineages of Pacific tree frogs may lead to behavioral reproductive isolation.

The causes of differences in the mating call among lineages of Pacific tree frogs are still unknown. However, interactions with the closely related California tree frog ( *P. cadaverina* ) offer a compelling hypothesis. Pacific tree frog mating calls from southern California have fewer pulses and slower pulse rates than calls of *P. cadaverina* (Littlejohn 1971; Straughan 1975). Straughan (1975) found that pulse rate was the only acoustic property used by female Pacific tree frogs to discriminate between conspecific mating calls and those produced by California tree frog males in Southern California. Since the range of *P. cadaverina* overlaps with the southern lineage of Pacific tree frogs, and since both species likely shared Pleistocene refugia during glacial maxima (Phillipsen and Metcalf 2009; Jadin et al. 2021), we propose that this is a case of reproductive character displacement. The split between California and Pacific tree frogs likely occurred during the Pliocene, approximately 3.5 million years ago (Phillipsen and Metcalf 2009; Jadin et al. 2021). As hypothesized in other systems (Höbel and Gerhardt 2003; Pfennig and Pfennig 2009), character displacement may have driven divergence in vocal sexual signals to minimize costly hybridization. While California and Pacific tree frogs may currently breed in different habitats (Phillipsen and Metcalf 2009; Stebbins and McGinnis 2011), we hypothesize that California tree frogs shared the same refugia and breeding habitats with populations of Pacific tree frogs resulting in the *hypochondriaca* lineage, driving more dissimilar vocal sexual signals, particularly for calls with slower pulse rates in Pacific tree frogs. This kind of interspecific signal divergence driven by historical sympatry has also been documented in Neotropical *Physalaemus* frogs (Boul et al. 2007), African reed frogs, *Hyperolius* (Bell and Zamudio 2012), and Amazonian *Hypsiboas* (Funk et al. 2012).

The consequences of the differences in the mating call between mitochondrial lineages of Pacific tree frogs are unknown. The extent to which females discriminate between lineages is still an open question, but the large differences in pulse rate suggest some degree of premating behavioral isolation. Previous studies have shown that pulse rate is a static property in Pacific tree frogs (Vélez and Guajardo 2021; Messersmith et al. 2024), and static call properties are under strong stabilizing selection and function in species recognition (Gerhardt and Huber 2002). Given the stark differences in pulse rate between lineages, it is plausible that females prefer calls of their own lineage. If such preferences exist, they may maintain lineage divergence even in zones of secondary contact. Thus, to start to understand the implications of between‐lineage call differences for mating behavior, and to better inform the taxonomy of this group, future studies should determine the extent to which female Pacific tree frogs show preferences for calls of their own lineage. If such preferences exist, future studies should also investigate the acoustic properties that mediate lineage‐specific preferences, and the shape (e.g., stabilizing vs. directional) and strength of those preferences.

Pacific tree frogs have been used as a model system in studies of anuran auditory physiology for decades (Straughan 1975; Alder and Rose 1998; Edwards et al. 2002). Neurons responsive to a species‐specific number of pulses—known as interval‐counting neurons—were first identified in this species (Alder and Rose 1998; Edwards et al. 2002). These “pulse‐integrator” or “interval‐counting” neurons have now been found in several species of frogs (Alder and Rose 1998; Edwards et al. 2002; Gupta et al. 2021). Importantly, these neurons show strong selectivity for the species‐specific pulse rate, and the minimum number of pulses necessary to elicit a response varies between species depending on the number of pulses in the mating call (Alder and Rose 1998; Edwards et al. 2002; Gupta et al. 2021). The tuning of these neurons depends on patterns of excitatory and inhibitory inputs (Rose 2018; Alluri et al. 2021), and we are starting to learn about the potential genetic mechanisms underlying differences in tuning properties of these neurons (Ospina et al. 2021; Anderson 2024). As such, between‐lineage divergence in call structure could reflect (or even drive) differences in neural tuning, which may be genetically based (Ospina et al. 2021). This makes Pacific tree frogs an ideal system for linking behavioral, neural, and genetic mechanisms of reproductive isolation.

Sexual signals are arguably one of the most important traits for the maintenance of premating reproductive isolation. Our study adds to growing examples of cases in which among‐population variation in sexual signals cannot be fully explained by random processes. Furthermore, we show here that properties of vocal sexual signals often used for species recognition vary strongly between mitochondrial lineages of Pacific tree frogs. The causes and consequences of such variation are currently unknown. Understanding the causes and consequences of such variation will help clarify the evolutionary forces contributing to the diversification and taxonomy of Pacific tree frogs.

## Author Contributions

**Alejandro Vélez:** conceptualization (lead), data curation (equal), formal analysis (equal), investigation (equal), visualization (equal), writing – original draft (lead), writing – review and editing (equal). **Colleen M. Ingram:** conceptualization (supporting), data curation (equal), formal analysis (equal), investigation (equal), visualization (equal), writing – original draft (supporting), writing – review and editing (equal).

## Conflicts of Interest

The authors declare no conflicts of interest.

## Supporting information


**Appendix S1:** ece372292‐sup‐0001‐AppendixS1.zip.

## Data Availability

All the required data are uploaded as Supporting Information and sequences generated in this study have been deposited to the NCBI GenBank database under accession numbers PV685967–PV685997.

## Appendix

**TABLE A1:** Bioclimatic variables and elevation of the nine localities (listed by county name) obtained from the WorldClim database (https://www.worldclim.org/; Hijmans et al. 2005) at a spatial resolution of 2.5′ arcminutes (~5 km^2^). Temperatures are provided in°K and precipitation values are adjusted by adding 1. For more information about the variables, readers are referred to Hijmans et al. (2005; https://www.worldclim.org/).

	Humboldt	Mendocino	Sierra	Lake	Orinda	San Luis Obispo	Santa Barbara	Los Angeles	San Diego
BIO‐01: Annual mean temperature	285.0	284.6	279.2	287.3	287.7	286.9	288.6	289.4	290.4
BIO‐02: Mean diurnal range	281.6	286.9	287.2	288.6	285.0	284.8	289.8	286.3	284.4
BIO‐03: Isothermality	56.1	54.1	43.0	48.6	51.6	65.0	58.3	52.8	55.3
BIO‐04: Temperature seasonality	244.5	441.9	664.6	616.2	431.8	224.5	416.5	426.8	336.4
BIO‐05: Max temperature of warmest month	292.8	299.8	298.6	306.0	300.5	296.0	304.7	303.8	300.5
BIO‐06: Min temperature of coldest month	277.7	274.3	265.8	274.3	277.5	278.0	276.1	278.8	280.0
BIO‐07: Temperature annual range	15.1	25.5	32.7	31.7	23.0	18.0	28.6	25.0	20.4
BIO‐08: Mean temperature of wettest quarter	282.5	280.2	271.8	280.5	282.4	284.5	284.3	285.1	286.9
BIO‐09: Mean temperature of driest quarter	287.8	289.9	287.3	295.0	292.4	289.1	293.4	294.3	294.1
BIO‐10: Mean temperature of warmest quarter	288.1	290.4	287.9	295.2	293.0	289.6	294.1	295.3	294.9
BIO‐11: Mean temperature of coldest quarter	282.2	279.7	271.8	280.5	282.4	284.3	283.9	284.9	286.6
BIO‐12: Annual precipitation	1206.0	1614.0	1127.0	891.0	702.0	548.0	483.0	427.0	318.0
BIO‐13: Precipitation of wettest month	208.0	302.0	198.0	188.0	152.0	116.0	100.0	92.0	64.0
BIO‐14: Precipitation of driest month	5.0	5.0	11.0	3.0	2.0	2.0	1.0	2.0	2.0
BIO‐15: Precipitation seasonality	79.9	88.0	74.0	91.9	93.6	96.0	95.9	99.9	88.2
BIO‐16: Precipitation of wettest quarter	599.0	852.0	534.0	485.0	387.0	323.0	282.0	247.0	177.0
BIO‐17: Precipitation of driest quarter	33.0	28.0	52.0	13.0	9.0	6.0	4.0	5.0	7.0
BIO‐18: Precipitation of warmest quarter	45.0	48.0	55.0	22.0	14.0	15.0	12.0	10.0	13.0
BIO‐19: Precipitation of coldest quarter	570.0	831.0	534.0	485.0	387.0	307.0	262.0	246.0	166.0
Elevation	35.0	572.0	1999.0	591.0	225.0	102.0	373.0	462.0	94.0

**TABLE A2:** GenBank accession numbers, individual identifiers used in the phylogeny, species names, and associated metadata for cytochrome *b* sequence data. The dataset includes curated sequences of *Pseudacris* species, with representatives from all recognized species, with an emphasis on the *Pseudacris regilla* complex and its sister taxa.

Individual ID	Species	Collection number	GenBank accession number	Original study
Outgroup	*Acris gryllus*	TG00016/ECM0052	EF988155	Gamble et al. (2008)
1	*P. crucifer*	JFBM14294	EF988160	Gamble et al. (2008)
2	*P. ocularis*	ECM0045	KJ536192	Barrow et al. (2014)
3	*P. ornata*	ECM0033/KU288911	KJ536205	Barrow et al. (2014)
4	*P. illinoensis*	ECM4370	KJ536208	Barrow et al. (2014)
5	*P. streckeri*	P3	KJ536206	Barrow et al. (2014)
6	*P. brimleyi*	ECM0469/TNHC6357	KJ536212	Barrow et al. (2014)
7	*P. brachyphona*	ECM0111	KJ536210	Barrow et al. (2014)
8	*P. clarkii*	ECM2478	KJ536214	Barrow et al. (2014)
9	*P. maculata*	ECM0099	KJ536217	Barrow et al. (2014)
10	*P. nigrita*	ECM0372	KJ536228	Barrow et al. (2014)
11	*P. fouquettei*	ECM0029	KJ536226	Barrow et al. (2014)
12	*P. kalmi*	ECM0162	KJ536222	Barrow et al. (2014)
13	*P. triseriata*	ECM3468	KJ536224	Barrow et al. (2014)
14	*P. feriarum*	ECM0448	KJ536220	Barrow et al. (2014)
15	*P. cadaverina*	ECM0150	KJ536194	Barrow et al. (2014)
16	*P. cadaverina*	H46	FJ599839	Phillipsen and Metcalf (2009)
17	*P. cadaverina*	H33	FJ599862	Phillipsen and Metcalf (2009)
18	*P. cadaverina*	H4	FJ599871	Phillipsen and Metcalf (2009)
19	*P. cadaverina*	H36	FJ599860	Phillipsen and Metcalf (2009)
20	*P. cadaverina*	H55	FJ599847	Phillipsen and Metcalf (2009)
21	*P. cadaverina*	H54	FJ599848	Phillipsen and Metcalf (2009)
22	*P. cadaverina*	H29	FJ599866	Phillipsen and Metcalf (2009)
23	*P. cadaverina*	H20	FJ599869	Phillipsen and Metcalf (2009)
24	*P. cadaverina*	H19	FJ599867	Phillipsen and Metcalf (2009)
25	*P. regilla*		AY363206	Ripplinger and Steven Wagner (2004)
26	*P. regilla*		AY363207	Ripplinger and Steven Wagner (2004)
27	*P. regilla*		AY363192	Ripplinger and Steven Wagner (2004)
28	*P. regilla*		AY363191	Ripplinger and Steven Wagner (2004)
29	*P. regilla*		AY363183	Ripplinger and Steven Wagner (2004)
30	*P. regilla*		AY363198	Ripplinger and Steven Wagner (2004)
31	*P. regilla*		AY363189	Ripplinger and Steven Wagner (2004)
32	*P. regilla*		AY363217	Ripplinger and Steven Wagner (2004)
33	*P. regilla*		AY363215	Ripplinger and Steven Wagner (2004)
34	*P. regilla*		AY363199	Ripplinger and Steven Wagner (2004)
35	*P. regilla*		AY363200	Ripplinger and Steven Wagner (2004)
36	*P. regilla*		AY363213	Ripplinger and Steven Wagner (2004)
37	*P. regilla*		AY363210	Ripplinger and Steven Wagner (2004)
38	*P. regilla*		AY363211	Ripplinger and Steven Wagner (2004)
39	*P. regilla*		AY363216	Ripplinger and Steven Wagner (2004)
40	*P. regilla*		AY363188	Ripplinger and Steven Wagner (2004)
41	*P. regilla*		AY363196	Ripplinger and Steven Wagner (2004)
42	*P. regilla*		KJ536195	Barrow et al. (2014)
43	*P. regilla*		AY363194	Ripplinger and Steven Wagner (2004)
44	*P. regilla*		AY363193	Ripplinger and Steven Wagner (2004)
46	*P. sierra*	SHL02		This study
47	*P. sierra*	SHL01		This study
48	*P. sierra*	SHL03		This study
49	*P. sierra*	SHL04		This study
50	*P. sierra*		DQ195201	Recuero et al. (2006)
51	*P. sierra*		DQ195203	Recuero et al. (2006)
52	*P. sierra*	EBMUD03		This study
53	*P. sierra*	EBMUD04		This study
54	*P. sierra*	MCL04		This study
55	*P. sierra*	MCL03		This study
56	*P. sierra*		DQ195207	Recuero et al. (2006)
57	*P. sierra*		DQ195199	Recuero et al. (2006)
58	*P. sierra*		KJ536201	Barrow et al. (2014)
59	*P. sierra*		AY363202	Ripplinger and Steven Wagner (2004)
60	*P. sierra*		AY363205	Ripplinger and Steven Wagner (2004)
61	*P. sierra*		AY363204	Ripplinger and Steven Wagner (2004)
62	*P. sierra*		DQ195200	Recuero et al. (2006)
63	*P. sierra*		KJ536202	Barrow et al. (2014)
64	*P. sierra*	MCL05	PV685981	This study
65	*P. sierra*	MCL01	PV685979	This study
66	*P. sierra*	ACRR04	PV685973	This study
67	*P. sierra*		DQ195206	Recuero et al. (2006)
68	*P. sierra*	HBNWR05	PV685969	This study
69	*P. sierra*	ACRR02	PV685971	This study
70	*P. sierra*	ACRR01	PV685970	This study
71	*P. sierra*	ACRR03	PV685972	This study
72	*P. sierra*	ACRR05	PV685974	This study
73	*P. sierra*		KJ536203	Barrow et al. (2014)
74	*P. sierra*	HBNWR01	PV685967	This study
75	*P. sierra*	HBNWR04	PV685968	This study
76	*P. sierra*	HBNWR02	PV685968	This study
77	*P. sierra*	HBNWR03	PV685968	This study
78	*P. hypochondriaca*		DQ195181	Recuero et al. (2006)
79	*P. hypochondriaca*	SEDG05	PV685990	This study
80	*P. hypochondriaca*	SEDG01	PV685988	This study
81	*P. hypochondriaca*	KNRM01	PV685984	This study
82	*P. hypochondriaca*		DQ195179	Recuero et al. (2006)
82	*P. hypochondriaca*	SEDG06	PV685991	This study
83	*P. hypochondriaca*		KJ536199	Barrow et al. (2014)
84	*P. hypochondriaca*	SRLA04	PV685994	This study
85	*P. hypochondriaca*		DQ195182	Recuero et al. (2006)
86	*P. hypochondriaca*		DQ195186	Recuero et al. (2006)
87	*P. hypochondriaca*	SRLA05	PV685993	This study
88	*P. hypochondriaca*	SRLA03	PV685993	This study
89	*P. hypochondriaca*	DLM04	PV685997	This study
90	*P. hypochondriaca*	DLM05	PV685997	This study
91	*P. hypochondriaca*		DQ195183	Recuero et al. (2006)
92	*P. hypochondriaca*		DQ195197	Recuero et al. (2006)
93	*P. hypochondriaca*	DLM03	PV685996	This study
94	*P. hypochondriaca*	SRLA01	PV685992	This study
95	*P. hypochondriaca*	DLM01	PV685995	This study
96	*P. hypochondriaca*		DQ195202	Recuero et al. (2006)
97	*P. hypochondriaca*		DQ195198	Recuero et al. (2006)
98	*P. hypochondriaca*		DQ195204	Recuero et al. (2006)
99	*P. hypochondriaca*	KNRM03	PV685985	This study
100	*P. hypochondriaca*	SEDG03	PV685989	This study
101	*P. hypochondriaca*		DQ195205	Recuero et al. (2006)
102	*P. hypochondriaca*		DQ195180	Recuero et al. (2006)
103	*P. hypochondriaca*	KNRM05	PV685987	This study
104	*P. hypochondriaca*		DQ195196	Recuero et al. (2006)
105	*P. hypochondriaca*	KNRM04	PV685986	This study
106	*P. hypochondriaca*		DQ195175	Recuero et al. (2006)
107	*P. hypochondriaca*		DQ195176	Recuero et al. (2006)
108	*P. hypochondriaca*		DQ195177	Recuero et al. (2006)
109	*P. hypochondriaca*		DQ195185	Recuero et al. (2006)
110	*P. hypochondriaca*		DQ195169	Recuero et al. (2006)
111	*P. hypochondriaca*		DQ195170	Recuero et al. (2006)
112	*P. hypochondriaca*		DQ195173	Recuero et al. (2006)
113	*P. hypochondriaca*		DQ195171	Recuero et al. (2006)
114	*P. hypochondriaca*		DQ195189	Recuero et al. (2006)
115	*P. hypochondriaca*		DQ195188	Recuero et al. (2006)
116	*P. hypochondriaca*		DQ195190	Recuero et al. (2006)
117	*P. hypochondriaca*		DQ195192	Recuero et al. (2006)
118	*P. hypochondriaca*		DQ195193	Recuero et al. (2006)
119	*P. hypochondriaca*		DQ195194	Recuero et al. (2006)
120	*P. hypochondriaca*		DQ195187	Recuero et al. (2006)
121	*P. hypochondriaca*		DQ195191	Recuero et al. (2006)
